# ‘More like a partnership’: A qualitative evaluation of Communication Coaching for Sonographers (CCS) in obstetric ultrasound

**DOI:** 10.1177/1742271X241277127

**Published:** 2024-09-25

**Authors:** Essie Kaur, Jane Arezina, Louise Bryant, Kathryn Pollak, Rebecca Wallace, Gill Harrison, Ruth Bender Atik, Roxanne Sicklen, Jen Coates, Natasha Hardicre, Teresa Lardner, Karen Horwood, Hannah Beety, Jon Arnold, Judith Johnson

**Affiliations:** 1 University of Leeds, Leeds, UK; 2 Health Services Management Centre, University of Birmingham, Birmingham, UK; 3 Specialist Science Education Department (SSED), Leeds Institute of Cardiovascular and Metabolic Medicine, School of Medicine, University of Leeds, Leeds, UK; 4 Division of Psychological and Social Medicine, Leeds Institute of Health Sciences, University of Leeds, Leeds, UK; 5 Department of Population Health Sciences, Duke University School of Medicine and Duke Cancer Institute, Durham, NC, USA; 6 Royal Gwent Hospital, Aneurin Bevan University Health Board, Newport, UK; 7 The Society and College of Radiographers, London, UK; 8 City, University of London, London, UK; 9 The Miscarriage Association, Wakefield, UK; 10 Royal Free London NHS Foundation Trust and Barnet Hospital, Barnet, UK; 11 Bereavement Support and Volunteering, Sands, London, UK; 12 NHS England, Leeds, UK; 13 Public Health Commissioning and Operations, Fetal Anomaly Screening Programme, NHS England, Leeds, UK; 14 Independent Lay Expert, Leeds, UK; 15 Nest Independent Midwifery, Leeds, UK; 16 Tiny Tickers, Leeds, UK; 17 School of Psychology, University of Leeds, Leeds, UK; 18 Bradford Institute for Health Research, Bradford, UK; 19 University of New South Wales, Sydney, NSW, Australia

**Keywords:** Communication, coaching, breaking bad news, pregnancy, ultrasound, burnout

## Abstract

**Introduction:**

Sonographers are required to deliver unexpected news to expectant parents in real time during obstetric ultrasound scans. The complexity of these interactions requires sonographers to conduct the clinical task while communicating their findings and managing the expectant parent’s response within the designated appointment time. Communication coaching for sonographers (CCS) is a tailored intervention that has previously been associated with improvements in confidence and news delivery practice. The current study explored the views and experiences of sonographers who completed CCS to evaluate and inform future delivery of this intervention.

**Methods:**

Nine sonographers participated in semi-structured qualitative interviews after completing CCS. We analysed data using a Reflexive Thematic Analysis (RTA) approach.

**Results:**

Participants reported CCS to be valuable and informative. The key themes identified included (1) innovating the path: tailored and novel training for sonographers, (2) humanising care: honouring the self, service-users and relevant others in the delivery of compassion-focused care and (3) making space: considerations for successfully delivering coaching. Participants said the coaching provided practical suggestions and was experienced as a ‘safe space’ for reflective practice that helped to enhance their capacity to identify and respond to emotion in others. There were practical challenges to taking part in CCS and organisational factors could act as a barrier; managerial championing of the intervention was crucial to uptake and completion.

**Conclusions:**

Participants viewed CCS positively. To implement CCS, there needs to be organisational coordination. Further controlled studies will be needed to establish the effectiveness of CCS.

## Introduction

Ultrasound is a primary diagnostic tool used by practitioners to generate real-time results in obstetric healthcare settings. In some healthcare systems, such as the United Kingdom’s (UK) National Health Service (NHS), sonographers communicate findings to the expectant parent in real time during the scan.^1^ Unfortunately, complications are a common occurrence as one in six pregnancies involve miscarriage or stillbirth,^2,3^ and one in fifty present a variation from expected development.^4–6^ The practice of communicating ultrasound findings reflects global trends in expectant parents’ preferences for the immediate communication of results.^7,8^ However, the real-time delivery of ‘unexpected news’ can create uncertainty and potential distress for expectant parents, which presents a complex challenge for sonographers.^7^

Unlike many other clinical or medical interactions between providers and service-users, sonographers are required to conduct the clinical examination, formulate a diagnosis, provide feedback on their findings, and manage expectant parents’ responses simultaneously.^9,10^ This is an arduous and complex challenge, especially when sonographer lists are fully booked, and they usually have limited pre-scan information, no warning about what they might find during the scan and no time to prepare how to explain the findings.^9,11^ The challenging nature of these interactions requires sonographers to manage their demeanour; expectant parents monitor their practitioner’s body language and facial expressions for ‘clues’.^7,9^ The highly idiosyncratic nature of pregnancy complications means there is no single word, phrase or approach sonographers can use when relaying unexpected or uncertain findings. Perhaps unsurprisingly, expectant parents sometimes report negative experiences of receiving unexpected pregnancy findings which are identified via ultrasound scan.^7^ Some parents have described delays in receiving news, the use of insensitive language, ambiguous phrasing and technical jargon, which caused them to experience strong negative emotional reactions.^7^

While there are established frameworks available for other healthcare professionals to use when delivering news to patients, these do not encompass the unique challenge of delivering news in real time and do not translate easily to the obstetric scanning context.^12^ In response to this gap, a consensus framework was developed to support sonographers in this difficult role.^12^ The framework is called ASCKS (Avoid assumptions; Set up the scan; Clear, honest information; Kindness; Self-care).^12^ The ASCKS guidelines make specific phrasing suggestions to support sonographers to communicate honestly, using sensitive, neutral and clear terms.^12^ The ASCKS framework is endorsed by UK professional guidelines and policy and has influenced similar Australian guidelines.^1,13^

To support sonographers to communicate in line with the ASCKS framework, we adapted an evidence-based communication training approach, Communication Coaching,^14^ for suitability for sonographers.^15^ Communication Coaching provides one-to-one supportive feedback to healthcare professionals from their own recorded and transcribed patient consultations.^14^ It has previously been found to be effective in nurses, physicians, and physician assistants.^16–18^ The adapted version, Communication Coaching for Sonographers (CCS), retains the basic principles of the original Communication Coaching intervention; the main divergence is that the focus is on supporting sonographers to communicate in line with the ASCKS framework.^15^

CCS is the first tailored intervention designed to support sonographers with news delivery. Two other interventions have been evaluated which aim to support healthcare professionals in maternity settings to communicate news, but these are focused primarily on non-sonographer groups including physicians, nurses and midwives, and do not address the specific challenges of communicating in ultrasound settings.^19,20^ Furthermore, while UK studies indicate that most sonographers have received news delivery training in some form,^11,21^ these programmes have not been formally evaluated and often rely on didactic teaching, which is not in line with sonographers’ training preferences.^21^

We have now completed and reported the first evaluation of CCS.^22^ Participating in CCS was associated with significant improvements in objectively rated communication skills and increased confidence in news delivery.^22^ All participants said they would recommend CCS to other sonographers.^22^ However, to gain a deeper understanding of the experiences of sonographers who took part in CCS, identify potential mechanisms of action and strengths of this intervention, and how it could be improved in future, qualitative interview data was analysed from sonographers who completed all aspects of the CCS intervention and who described their experiences of participating.

## Methods

### Design

The study used a qualitative interview design guided by Reflexive Thematic Analysis (RTA), a theoretically flexible approach which aims to generate patterns of shared meaning, known as ‘themes’.^23^ RTA is commonly used in healthcare research as it can generate useful insights to inform practice. In the present study, the approach taken to RTA was inductive, directed by the content of the data; semantic, in that we focused on the explicit content of the data; and critically realist, assuming that the data reflected a reality filtered by the mind of the researchers.^23,24^

### Intervention

CCS is an adapted version of Communication Coaching, which aims to improve practitioner communication competence and confidence via supportive, one-to-one feedback on audio-recorded encounters with service-users.^15,22^ CCS comprises three 30-minute online sessions delivered via video-platform by a Communication Coach. The timing of these sessions varied between participants, depending on factors such as the frequency with which they scanned in the Early Pregnancy Unit (EPU), the frequency with which they delivered unexpected news, and whether they received time during work to take part in the coaching. In the present study the coach was JJ, a Clinical Psychologist who was trained in the coaching approach by KP, who is the original creator of Communication Coaching. The first CCS session is introductory; the ASCKS principles are outlined, and coach-recipient rapport is built. The second two sessions provide feedback on the recipient’s transcribed news delivery encounters. All feedback is positively oriented, with practitioner strengths recognised and reinforced and suggestions made for ‘tweaks’ which practitioners can make to further enhance their communication practices.^14^ In the present study, recordings were made in the EPU due to a higher overall rate of unexpected news delivery in this setting.

### Participants and recruitment

All participants were sonographers who completed CCS as part of a wider evaluation.^22^ Sonographers were eligible to participate in this wider evaluation if they were obstetric ultrasound practitioners based in participating hospital sites who held some sessions in their site’s EPU. Three hospital sites in England and Wales participated. Following the intervention and online quantitative data collection survey, we invited participants to a qualitative interview with the project’s Research Assistant (EK). Of the 10 participants who completed all elements of the coaching, 9 participated in the qualitative interviews. Sample size in qualitative research is a contentious topic, and some researchers recommend 12 as a sample size guideline.^25^ However, we were guided by the concept of ‘information power’.^26^ This concept focuses on the ‘power’ of the data to meet the objectives of the research question rather than the number of interviews per se, where the focus of a question is more restricted, and the relevant knowledge of the participants greater in relation to the question is great, fewer participants are needed. The focus of our question was specific (on experiences of an intervention) and the knowledge of participants was significant in relation to this, so we deemed nine interviews sufficient.

Participants included six sonographers, one senior sonographer, one advanced practitioner sonographer and one sonographer with specialist obstetric interest. The remaining eligible participants who did not provide interview data said they enjoyed CCS, but we were unable to schedule an interview with them; they did not provide a reason for this. All participants were women, White or White British, radiographers by background and aged between 30 and 50 (mean age = 39.00, SD = 3.94). Participants had been qualified between 2 and 20 years (mean time = 11.00, SD = 5.68).

### Data collection

Data collection occurred between August 2022 and June 2023. EK conducted in-depth semi-structured interviews via Microsoft Teams or Zoom. Informed consent was verbally captured using the digital platform’s screen recording and transcription functions. Interviews comprised of two parts: (1) open-end questions to gain descriptive and reflective data and (2) closed-end questions to gather quantitative data about participant’s experiences of the intervention. The current report analysed data from the first part of the interview schedule; data from part 2 has been reported elsewhere.^22^ The interviews followed a semi-structured approach, averaging 20 minutes and were audio-recorded and transcribed verbatim (Table 1).

**Table 1.** List of questions and prompts used for the interview schedule.

Key question	Probe questions
What was your overall perception of the coaching?	Probe length/amount of sessions Was three sessions altogether too few/too many? Were sessions too long/too short/about right?
	Probe content of sessions How was the intro session? Did it provide the necessary info? How did feedback on transcripts work in the second two sessions? How was the 1:1 approach? Would a group session/element of the coaching been helpful?
	Probe online approach to coaching – would in-person have been better/worse?
What did you think worked well?	Any aspects which should remain the same, if changes are made to the coaching?
	Anything particularly useful?
What could be improved?	Anything else you would have liked to see in the coaching which was not included?
	Anything you thought should be removed?
How did this contribute to your learning and overall professional development relative to communication and delivering unexpected news?	Anything you now do differently as a result of attending the training?

### Data analysis

We audio-recorded interview transcripts and transcribed them via the interview platform. Two researchers (EK and JA) independently analysed and coded transcripts inductively for evaluative themes from close reading and re-reading of the data.^27^ We modified and developed codes throughout the RTA coding stages and had team discussions to determine key ideas identified in the data about the coaching and sonographers’ experiences of applying their learning to practice.^23^ EK and JA expanded and/or reduced the final agreed codes into meaningful categories to create themes and sub-themes.

## Results

The main themes that were identified from the interviews were (1) innovating the path: tailored and novel training for sonographers, (2) humanising care: honouring the self, service user and relevant others in the delivery of compassion-focused care and (3) making space: considerations for successfully delivering coaching. The themes and sub-themes are presented with key ideas in Table 2. Common sentiments communicated included that delivering unexpected news was described as emotionally taxing and required complex management of clinical and interpersonal tasks. Overall, participants reported the coaching to be beneficial and worthwhile because it improved their confidence, interpersonal skills and ability to meaningfully engage with expectant parents and their partners by using the ASCKS framework in their clinical practice to deliver unexpected news in a structured manner.

**Table 2.** Themes and sub-themes with participant quotes and key ideas and concepts.

Themes	Sub-themes	Key Ideas/Concepts
1. Innovating the Path: Bespoke and Novel Training for Sonographers	1.1. Tailored approach to suit the needs and wants of the Sonographer	The coaching enabled sonographer-led learning and application of new skills and knowledge: Coaching informs preferences to styles of (i) communication and (ii) learning Provides sonographers with collaborative freedom with the coach based on own expertise and lived experiences Coach’s insights, expertise and recommendations were valuable and transferable Self-directed learning and high degree of choice for context-based application of skills and knowledge Provides objective framework to use as a baseline for structured and effective communication for all skill levels (experienced and newly qualified) Transcripts, framework and leaflets were valuable and objective resources
	1.2. A positive, supportive and reassuring learning process	Learning process honours sonographer’s comfort and support
	1.3. Flexible and structured approach with noticeable progression	The coaching suits differences in working schedules and lifestyles, in a manner that is well-paced and enables recognisable progression: The course of one-to-one sessions complimented working schedules and alleviated pressures with adequate time in between sessions to apply learning Sonographers recognised learning, adoption of skills and improvement during and completing the coaching
	1.4. Champions continuing professional development	Focuses on the patient-centred approach Recognised significance and benefit to clinical practice
	1.5. Aligns with wider NHS values and goals	Corresponds with NHS goals of compassionate and patient-focused care
2. Humanising Care: Honouring the self, patient and relevant others in the delivery of compassion-focused care	2.1. Reflection and perspective expansion	The coaching and resources encourage reflective practice and opportunities to capitalise on own experiences and expertise in a collaborative manner with the coach that is relatable to sonographers: Fosters the importance of the Self in delivering news (expression, esteem and confidence) Recognising differences in the delivery of care between others including (i) Patients and (ii) Colleagues Cultivates expansive thinking to consider other’s point of view in the delivery of unexpected news and across clinical scenarios
	2.2. Recognising and responding to emotions	Sonographers reported an increased ability to better recognise, name and respond to emotions during interactions with patients: Cultivating a human response during difficult interactions Receiving and providing clear, honest and empathic communication The ASCKS framework makes it easier to navigate communicating and interacting with others during emotional scenarios Dynamic use of skills to execute the clinical task and attend to patients needs
	2.3. Meaningful connectivity with others	Recognition of better interpersonal skillset and capacity Newfound appreciation, understanding and recognition of and with others Fosters connectivity between colleagues, patients and stakeholders
3. Making Space: Considerations for successfully delivering coaching	3.1. Expectations, Attitudes and People	Managing negative/inaccurate expectations of coaching and improving unexpected news delivery Champion the reality of positive experiences of coaching and making the delivery of unexpected news better
	3.2. Capacity and Co-production	Co-production to implement coaching and mitigate barriers between colleagues and departments Team and Manager support are instrumental (organisational culture and climate) Preferred Online Modality

NHS: National Health Service; ASCKS: Avoid assumptions; Set up the scan; Clear, honest information; Kindness; Self-care.

### Innovating the path: tailored and novel training for sonographers

All participants detailed the tailored approach of the coaching as innovative and complemented by the structured, well-paced and flexible delivery of coaching, which honoured the sonographers’ schedules and capacity. Some thought that the coaching addressed a current training gap in the profession and one which could have significant impact on clinical training.

#### Tailored approach to suit the needs and wants of the sonographer

Participants provided positive feedback on how the content, recommendations, and one-to-one approach were individually tailored. The coach’s psychological expertise and guidance were reported as instrumental when reviewing transcripts from their own scanning sessions and helped them to identify areas of communication that could be ‘tweaked’. From their one-to-one sessions, participants said they were able to determine their preferences and capitalise on their strengths in communication and delivering unexpected news using the ASCKS framework. The transcript-based recommendations enabled them to adopt and practise skills during subsequent scanning sessions and identify what suggestions worked best for them. This freedom to practise and naturally apply elements of the ASCKS framework was a commonly reported factor that was thought to improve sonographers’ abilities to deliver unexpected news during scans:

*To have somebody like [the coach], who’s obviously got a lot of knowledge from a psychology point of view, [their] interpretation and sort of viewpoint on what you’ve said and what was good and what was bad I guess this is something we’ve never encountered before. I’ve never had like as part of our training, we don’t really. There isn’t a psychology element to it particularly. I’ve never kind of had that other than my own colleagues. I’ve never had the input from another sort of professional on what’s going on or what could be improved. (P4)*

Participants felt the coaching experience was a valuable addition to their practice, and they were able to direct their own learning. The coaching sessions allowed a safe platform for critical discussion of what did and did not work for them, with some describing certain elements of the framework to be interchangeable to suit the context. The additional accessibility of tools and resources helped to adopt the ASCKS framework, which all participants described as an objective baseline for structured and effective communication in clinical practice:

*Some things that we discussed whether we thought would work or wouldn’t work or again, would depend on the situation or I felt for me personally that perhaps wouldn’t come naturally from me, and obviously I want to always be sincere with patients rather than sound contrived (P9)*

*I thought the coaching was great. Really informative. [the coach] was absolutely lovely, really talked you through it and the little leaflet thing that we got given as well with all the pointers on it. I did think it was very well formatted out. Yeah, I thought it was great . . . it was all very clear, concise and I loved that kind of approach (P11)*

#### A positive, supportive and reassuring learning process

All participants detailed their experience as positive, supportive and reassuring learning process, in which they felt they could be comfortable, vulnerable, and honest with the coach. Some participants identified that many sonographers are fearful of criticism and scrutiny from others about their competence and practice. Participants felt the supportive and non-judgemental approach was empowering and a much-needed addition to their typical practice:

*I kind of thought it would be a little bit cringey if I’m honest, sort of reading the things you’d read you’d said and maybe hearing your own voice would be too cringy. I think it’s better to do it as a transcript rather than an audio recording. But actually, I didn’t feel like that because it was obviously really positive and it was like I like ‘what you’ve said here’ and ‘I like that you’ve said this and maybe you could keep this little bit’ so I feel like looking at the transcripts was really useful. (P4)*

#### Flexible and structured approach with noticeable progression

Participants felt the flexible scheduling of sessions and access to resources complemented their working schedules and was a rare, yet necessary change for sonographers working in NHS organisations. They explained that they are usually limited to courses provided by external organisations or further academic training. They must attend these out of working hours, most of which are objectively delivered without one-to-one support based on sonographer’s own experiences. The structured and flexible approach allowed the participants to develop their technique and skills in a manageable fashion that accommodated both their working schedules and lifestyles:

*The sessions I didn’t think they were too arduous or long winded. And I think it was nice to go through a couple of times absolutely just to see if you’re, you know, improving your technique. I thought it was, yeah, well set out. (P11)*

All participants detailed accounts of recognised learning, adoption of skills and improvement during and after completion of the coaching. Both newly qualified and experienced sonographers described noticeable progression in their communication and changes in their practice. The time to think and apply skills between sessions was an essential element of learning transfer:

*Those sessions in particular I found really helpful because I did learn a lot from them, even though I’m very experienced in sonography, I think it was really useful to have recommendations of how to change practice. (P1)*

*I thought it was very well organised and everything that was given to us I felt was given to us in bitesize manageable portions so that we could implement that before going on to the next step. . . I do believe that it has improved my practice. (P7)*

#### Champions continuing professional development (CPD)

Some participants felt the coaching encouraged reflective practice and a means for CPD. The process provided ample opportunity to collaboratively discuss and be intentional about their developmental needs, which they felt they did not usually get enough time for. This dedicated time for in-depth reflection provided a space to refocus and reconnect to the purpose of their practice and acted as a catalyst to implement change, as well as opportunities to recognise subsequent improvements in their communication. All participants felt it would be useful to sonographers, irrespective of their level of expertise and experience. Some expressed that the coaching was a good refresher for maintaining good clinical practice:

*It was really helpful and useful to be able to sort of word-by-word see what you’d said because obviously as healthcare professionals, we do try to reflect on our practice, but often it’s just a sort of skimming over what went well or went badly, and if I ‘m honest, I don’t do that for every patient. Obviously because you just do not have time and even if you do, you cannot remember some of the subtleties that you might have said or the tone of voice you might be used. So, it’s really useful to go over that in such detail and then to have somebody, in a nice way, dissect it as well, it is really really useful and I felt that. (P4)*

*I do think it’s something that all sonographers should do, I do think I have improved. It made me feel more confident doing it and made me feel like I’m way there is more sort of patient focused. So yeah, definite improvement. (P11)*

#### Aligned with wider NHS values and goals

The coaching was described as meeting the wider departmental and NHS values and goals. Some participants described how the coaching championed the patient-centred approach while respecting the sonographer’s personal experiences and challenges. Often, the understanding of delivering patient-centred care is not effectively translated into practice but the ASCKS framework provided sonographers with the confidence to communicate in a clear, concise, and compassionate manner, while enabling them to feel prepared when delivering unexpected news:

*I found it relevant to clinical practice and I thought it was a really good way to refocus and reconnect. (P13)*

*But I think, you know the patient-centred approach, it’s kind of thrown around a lot as a thing and I don’t always feel really get what it means. It just kind of say it to sound good and it feels like it actually is at the heart of what you’re doing, which is lovely. (P6)*

### Humanising care: honouring the self, service user and relevant others in the delivery of compassion-focused care

Some participants said that the intervention contributed to their reflections on their practice and development and expanded their knowledge and skills in a relational sense.

#### Reflection and perspective expansion

Participants reported the coaching encouraged reflective practice because it provided opportunities to reflect on their own clinical experiences and expertise. These reflective discussions enabled them to consider expectant parents’ points of view in the delivery of unexpected news across clinical scenarios. Some felt the dedicated support and guidance encouraged them to consider the importance and impact of their role and behaviour when delivering unexpected news, which contributed to increased emotional expression, self-esteem, and confidence:

*It felt more like a partnership. (P4)*

*The additions that [the coach] was putting on my transcripts were totally, you know, good ideas and somethings that we discussed whether we thought would work or wouldn’t work or again would depend on the situation or I felt for me personally. (P9)*

*I like the one-to-one. It was good. It gave us an opportunity to sort of like talk about different things as we were going on and reflect on, not just scenarios that we’ve been through with these three coaching sessions, but others you know sort of in that we’ve gone through, I’ve come across in the past and different things. (P15)*

#### Recognising and responding to emotions

All participants believed they could better recognise and respond to emotions during interactions with expectant parents following the coaching, which enabled them to navigate the conversations in a constructive yet compassionate manner. The ASCKS framework provided a solid baseline with interchangeable phrases that sonographers could use to suit the context, and this made them feel well-equipped to respond in a considerate and empathetic manner during difficult interactions. Notably, sonographers reported this element of their training was transferable to other situations when communicating with patients, colleagues and others:

*Now I’m a lot more conscious of naming the emotion that the patient is experiencing and being comfortable doing that. I think in the past I either didn’t want to name it, because I was worried I was going to get it wrong, or you want to name it because it’s like the elephant in the room and you kind of don’t want to. (P4)*

#### Meaningful connectivity with others

Participants felt that the coaching helped them develop their ability and capacity to meaningfully relate to others. Many recognised positive and valuable changes in their interactions, which made them feel more confident and focused on their tasks and the wellbeing of service-users. This made delivering unexpected news feel less burdensome and taxing and allowed participants to foster sincere connectivity between colleagues, service-users and stakeholders towards positive and sensitive communication that did not feel contrived:

*I find it really valuable to my practice and listening to colleagues talking, I’ve kind of felt that it would be beneficial to all sonographers and not just me. (P1)*

*It’s really useful and it has made a difference to how I interact with people like some of the words I say. (P6)*

*So for the one patient I did manage to get it was quite nice at the end for them to actually say thank you back. And I’d never really appreciated how much a thank you means or the fact that they went out of their way to say thank you in the environment and the situation that they were in. So I don’t know if I would have achieved that without the coaching sessions. (P7)*

### Making space: considerations for successfully delivering coaching

Across each hospital site, the personnel and capacity within the individual departments played an integral role in the success of CCS’s uptake and completion. The success of the CCS’s implementation was attributed to supportive and encouraging teams and senior staff, particularly managers. From a practical point of view, consideration of department capacity and willingness to collaborate played an essential role in being able to engage with the intervention effectively. Many discussed barriers and considerations to improving the uptake of CCS.

#### Expectations, attitudes, and people

Many participants expressed that they had concerns and negative expectations prior to taking part in CCS, which they said presented an embedded barrier to intervention uptake. However, the participants felt that the reality was empowering and positive, challenging negative and inaccurate expectations of what CCS would involve:

*I think we criticize ourselves more harshly than what we would other people. (P2)*

*I feel like some of my colleagues. . . they don’t see its value, or perhaps feel vulnerable about going into that [the coaching] . . . I think it’s really difficult because it’s the heart of it, I feel like it’s changing attitudes and members of staff. (P6)*

#### Capacity and coordination

CCS required coordination between participants, other sonographers, managers, and the coaching team to be successfully implemented. Some participants felt their wider team did not value or recognise the benefit of improving unexpected news delivery, which made others less inclined to participate. Some felt their senior staff needed to champion CPD and the type of support embedded within the coaching. Managerial support was described as a key factor in uptake of, and retention within, the intervention. Participants highlighted how organisational culture and climate may also influence whether a department can or will accommodate training for their staff:

*As I said when I started this, we were never actually given any official sort of training in how we should break bad news. It’s just things that you’ve developed over the years and you never know whether what you’re doing is right. So it’s nice for somebody to cause we don’t have people in with us either, so it’s nice for somebody to actually listen to how we’re saying and communicating things with patients and give us the feedback they actually know you are doing a good job. You know, there’s a few tweaks that maybe you could do, but overall, know what you’re doing is fine. So it is nice to have that. (P15)*

A well-appreciated factor of CCS was its online delivery which made it easier to accommodate it into busy working schedules. All participants agreed that the online modality made the coaching experience easy and accessible. The addition of an individual or group face-to-face session with the coach was commonly suggested to further improve engagement:

*I was quite happy with online and actually I think we’ve all got used to that sort of thing now as well. . . And it actually allowed me to fit it into a very busy schedule, so I didn’t have to take out a lot of time. For travelling or getting somewhere or expecting somebody to see me or even organise two or three of us to be off at the same time. Whereas I could manage 40 minutes or an hour within a schedule. And again, I could manage it for others to have that time off. So, I was very happy with the online aspect to it. (P7)*

*I think online it’s much easier to sort of implement with how busy and everything that we are at the moment. You know, being able to sort of earmark sort of half an hour here and there was much more easily, easily accommodatable than to try and organise something face to face. So yeah. (P15)*

## Discussion

The present study reports on the first qualitative evaluation of a communication training intervention designed specifically for sonographers working in obstetric ultrasound. The tailored, bespoke, and flexible nature of CCS was welcomed by participants, who felt it was effective in helping them to improve their news delivery practices and build their confidence. In doing so, participants felt it provided for their own professional development needs but also helped to meet wider health system goals by improving their capacity for patient-centred care. Beyond this, participants described developing a wider ability to recognise and respond to emotion. Participants also identified complexities in participating in CCS, outlining a need for cooperation and coordination between their manager, their team and the coach. They suggested that managers needed to champion CCS in their department for them to be able to take part and that a lack of support could prevent some from being able to take up CCS.

A previous study reported on the quantitative data generated from the evaluation.^22^ Objective ratings indicated that CCS was associated with increases in communication skills, and subjective reports indicated that CCS was associated with increased communication confidence in sonographers.^22^ Participants in the study also provided positive quantitative feedback, with the majority describing CCS as engaging and all stating they would recommend it to others.^22^ The present study generates insights regarding the mechanisms underlying these findings. Key elements identified as leading to change by participants included (1) the provision of clear, actionable, and credible recommendations, (2) the creation of a positive coach-recipient relationship to build confidence and enthusiasm in practice and (3) the coaching being a ‘safe space’ to reflect on performance and identify ways to improve without fear of criticism.

The quantitative evaluation also identified a moderate drop-out rate in participants; although 15 sonographers signed up for CCS, only 10 received all elements of the coaching.^22^ The present study suggests that attrition rates might be attributable to challenges in implementing the intervention in NHS settings and training for sonographers. While CCS was experienced positively, participants noted that several parties needed to cooperate to deliver CCS, including themselves, their manager and team, and the coaching team. Managers were identified as important influencers. Participants also identified wider cultural considerations which could prevent the successful implementation of CCS, including fear of criticism by the coach, which they believed were unfounded after participating in the intervention. These findings suggest that it is unlikely that the drop-out was due to negative experiences of the intervention and rather due to organisational and cultural barriers to participation.

Several studies have now evaluated Communication Coaching in a range of settings and healthcare professional groups, including nurses, doctors, and physician assistants, with positive results.^14,16,17^ The present study extends this by evaluating Communication Coaching in sonographers and finding further supportive evidence for the value of this intervention. This is also the first study to take a qualitative approach to evaluating Communication Coaching. Key elements hypothesised to be important in the implementation and effectiveness of the intervention include (1) the building of positive rapport between coach and recipient, (2) a prioritisation of positive feedback to build confidence and (3) a focus on supporting clinicians to identify and respond to service user emotion.^14^ The present study identifies these as important elements according to coaching recipients, supporting the underpinning philosophy and principles behind the design of Communication Coaching.

### Strengths and limitations

The study benefitted from a strong research design involving in-depth one-to-one interviews, which allowed for the generation of detailed data. It was limited by a gender imbalance in participants, with no male representation, but this reflects the ultrasound workforce, which includes a high proportion of women. Furthermore, all participants were White; future studies should include participants from a wider range of ethnic minority backgrounds. The largest limitation of the study, however, was a small sample and moderate attrition rates within the intervention. While the attrition rate is similar to other preliminary studies of intervention in healthcare professionals,^28,29^ the small sample is likely attributable in part to the timing of the study, which was during the COVID-19 pandemic. It could also reflect a bias in recruitment towards sonographers who were inherently more motivated to participate. It is unclear how this may have impacted the qualitative results and a larger study is needed to establish this. Another important limitation is that both the previous quantitative evaluation of CCS^22^ and the present qualitative evaluation collected no data from expectant parents. Thus, while conclusions can be made regarding sonographer communication practices and sonographers’ experiences, it is currently unclear whether CCS impacts the patient experience.

### Implications

There is a clear need for better support for sonographers when communicating unexpected news to expectant parents. Previous news delivery frameworks and interventions have been designed for other healthcare professionals in less time-pressured contexts.^12^ Due to the immediate nature of communication in obstetric ultrasound settings, these are not adequate for the challenges faced by sonographers.^12^ The present study provides further supportive evidence suggesting that CCS would be well received by sonographers and could be a useful intervention to offer in practice. However, it should be noted that our sample size is small and further evaluation using a controlled design should be undertaken; as such, CCS could be considered a useful candidate intervention for further investigation in sonographers. Our findings also highlight the need for organisational support to successfully deliver and evaluate CCS and emphasise the key role of supportive management. As yet, no data has been collected from expectant parents regarding CCS; future studies should seek to explore whether CCS can improve their experiences of receiving unexpected news which is identified during the ultrasound scan.

## Conclusion

Delivering unexpected news to expectant parents is a challenging task for sonographers, and better evidence-based support is needed. CCS is the first tailored intervention designed specifically to support sonographers with this task. CCS is well received by sonographers and helps them to provide patient-centred care.
